# Next-generation fluorescent nucleic acids probes for microscopic analysis of intracellular nucleic acids

**DOI:** 10.1186/s42649-019-0017-1

**Published:** 2019-11-18

**Authors:** Akimitsu Okamoto

**Affiliations:** Research Center for Advanced Science and Technology, The University of Tokyo, 4-6-1 Komaba, Meguro-ku, Tokyo, 153-8904 Japan

**Keywords:** Methylated DNA, RNA, Fluorescence, Single cell imaging, Synthetic nucleic acid

## Abstract

Fluorescence imaging of nucleic acids is a very important technique necessary to understand gene expression and the resulting changes in cell function. This mini-review focuses on sequence-specific fluorescence imaging of intracellular RNA and methylated DNA using fluorescent nucleic acid probes. A couple of functional fluorescent nucleic acid probes developed by our laboratory are introduced and the examples of their application to fluorescence imaging of intracellular nucleic acids are described.

## Introduction

The mechanism of gene expression in a various of cell events is the most important research target in molecular biology and cell biology researches. Imaging of these events within individual cells (Dirks and Tanke 2006; Silverman and Kool 2005), and even within organisms (Weigert et al. 2013), should be less invasive and more suitable for monitoring of intracellular events than the traditional biochemical methods, and thus, fluorescent signals are frequently used for monitoring of biomolecules and their functions in a cell. Visualizing the behavior of nucleic acids in cells is the best way to understand ongoing cellular function expression, and a number of fluorescent nucleic acid probes have been developed for sensing of target intracellular nucleic acids. Because hybridization of short fluorescent nucleic acids complementary to the target nucleic acid sequence is very sequence-specific, flexible design of fluorescent nucleic acid probes give us a direct method for the visualization of intracellular nucleic acids when the sequence information is available.

Design of ‘fluorescent nucleic acid probes’ which can be removed or turned off when the probe does not recognize its target nucleic acid is very important for the establishment of microscopic analysis of intracellular nucleic acids without a sensitive washing process, resulting in a highly reliable, labor-saving, and real-time fluorescence observation. The chemistry of sequence-specific fluorescence emission control is a significant key for the design of a next generation of functional fluorescent probes. Various DNA probes whose fluorescence intensity is controlled in a sequence-specific fashion have been widely used (Tyagi et al. 1998; Tyagi and Kramer 1996; Piatek et al. 1998; Fang et al. 2000; Broude 2002; Okamoto et al. 2003; Tan et al. 2004). However, many problems remain therein: (1) different types of dyes are required in a probe; (2) both strand ends are occupied by different dyes; and (3) probes require the formation of higher-order structures for efficient fluorescence quenching. Therefore, fluorescent nucleic acid probes using a new concept that solves these problems need to be developed to produce the next generation of fluorescent probes for nucleic acid sensing.

This mini-review focuses on sequence-specific fluorescence imaging of intracellular RNA and methylated DNA using fluorescent nucleic acid probes. A couple of functional fluorescent nucleic acid probes developed by our laboratory are introduced and the examples of their application to fluorescence imaging of intracellular nucleic acids are described. Their fluorescence emission was sequence-specifically controlled by the unique chemistry such as hybridization-sensitive fluorescence emission or methylation-specific metal complex formation.

## Live cell imaging of intracellular RNA

Nucleic acids labeled by thiazole orange dyes (TO) are well known to have strong hybridization-dependant fluorescence. However, the fluorescence suppression observed in the single-stranded state of TO-labeled DNA derivatives is still insufficient, and a more specific design is required for effective sensing of intracellular nucleic acids without background fluorescence.

**Fig. 1:**
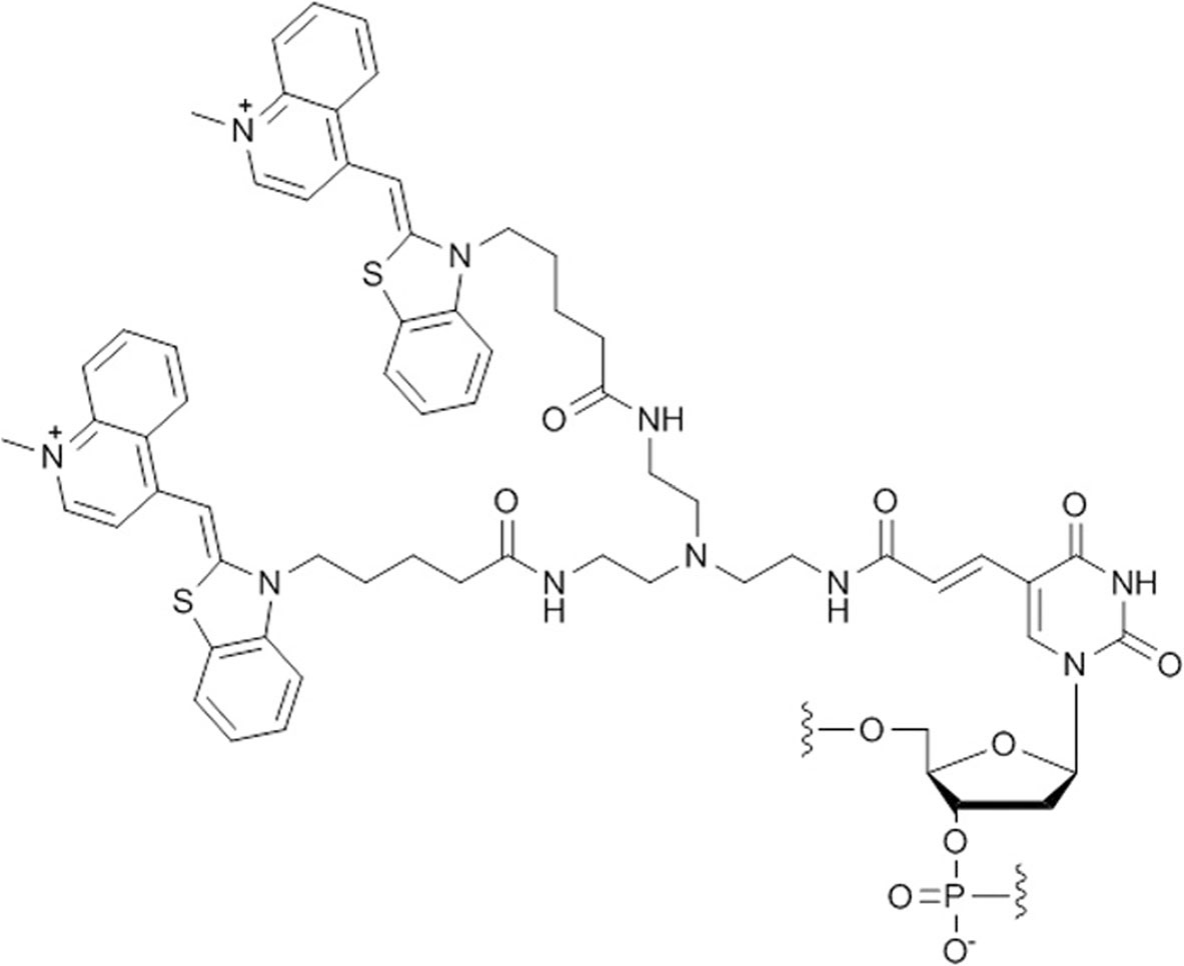
Doubly TO-labeled nucleotide “D_514_” in ECHO probe

We designed a DNA strand containing a nucleotide in which two TO subunits are linked covalently (Fig. 1) (Ikeda and Okamoto 2008). TO dyes are well-known for showing suppression of emission by the exciton coupling effect (Khairutdinov and Serpone 1997; Simon et al. 1998; Cosa et al. 2001; Sagawa et al. 2004; Fürstenberg et al. 2006) when they are arranged parallely (H-aggregation) (West and Pearce 1965; Czikkely et al. 1970; Harrison et al. 1996; Zeena and Thomas 2001; Hannah and Armitage 2004; Rösch et al. 2006). The excited state of the TO H-aggregate splits into two energy levels (Kasha 1963; Kasha et al. 1965; Levinson et al. 1957; McRae and Kasha 1958): The transition to the upper excitonic state is allowed for the H-aggregates, and the H-aggregates rapidly deactivate to a lower state. However, emission from the lower state is theoretically forbidden, and the H-aggregate is nonemissive. In the unhybridized state of the doubly TO-labeled DNA strand we synthesized, the blueshift of the absorption band of TO was observed, indicating excitation of H-aggregate of TO. The emission intensity was strongly suppressed. On the other hand, after hybridization of the doubly TO-labeled DNA strand with the complementary nucleic acid, strong emission was observed. The doubly TO-labeled DNA strand, in which the fluorescence emission is controlled by such excitonic interaction, was named the ECHO (exciton-controlled hybridization-sensitive fluorescent oligonucleotide) probe (Wang and Okamoto 2012; Okamoto 2011).

ECHO probe is very effective for sequence-specifically visualizing single-stranded nucleic acids, such as mRNA. Sequence-specific nucleic acid imaging has greatly advanced by the development of fluorescence in situ hybridization (FISH) methods (Nath and Johnson 2000; Levsky and Singer 2003; Smolina et al. 2007; Jaco et al. 2008), but conventional FISH is cumbersome and time-consuming, because it contains sensitive washing processes. On the other hand, ECHO probes have the higher sequence-specific fluorescence emission ability compared with conventional fluorescence-labeled nucleic acid probes, and thus a high-resolution FISH has become possible by adapting ECHO probes (ECHO–FISH) (Wang et al. 2012). ECHO–FISH is highly reproducible, stringent, and compatible with other fluorescent cellular labeling techniques. This method does not require stringent washing steps and it is accomplished in a 25-min procedure from fixation to mounting. The resolution allows detection of intranuclear speckles of poly(A) RNA in HeLa cells and dissociated hippocampal primary cultures, and mRNAs in the distal dendrites of hippocampal neurons.

The character of fluorescence switching by ECHO probes is suitable for visualizing mRNA localization in a living cell. The probes were introduced into cells by using manipulator-assisted microinjection, lipofection, or electropolation to visualize intracellular mRNA localization. For example, immediately after the injection of 5′-T_6_D_514_T_6_–3′ (D_514_ is a thymine derivative doubly labeled with two TO dyes) into the nucleus of a living HeLa cell, fluorescence was observed from the nucleus (Kubota et al. 2009a). ECHO probes with a DNA backbone are digested by intracellular nucleases and its half-life time is about 3 h in living cells. In order to add nuclease resistance to ECHO probes for the long-term intracellular RNA observation, the backbone of ECHO probes was replaced with a 2′-O-methyl RNA (Kubota et al. 2009b). The high nuclease resistance of new ECHO probes with 2′-O-methyl RNA makes long-term intracellular RNA imaging possible. Intracellular mRNA behavior during cell life events, such as migration and division, was observed through long-term monitoring of the cells into which a 2′-O-methyl RNA probe, 5′-^OMe^U_6_D_514_^OMe^U_6_–3′, was transfected. The fluorescence of the probe in the mother cells was evenly distributed to their two daughter cells after cell division.

**Fig. 2:**
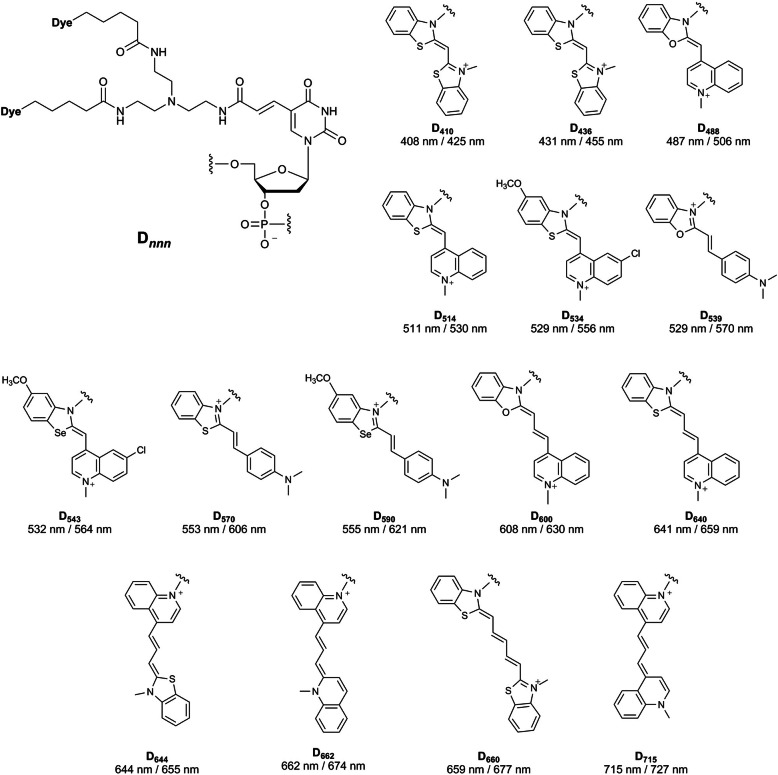
A series of doubly fluorescence-labeled nucleotides (D_*nnn*_) used in ECHO probe. Chemical structures of D_*nnn*_ are drawn with their excitation/emission maxima

A series of new ECHO probes in which the TO moiety was substituted by its derivatives (from blue to the near-infrared region) made it possible to understand spatiotemporal correlations between gene expression and the interaction of nucleic acids in living cells (Fig. 2) (Ikeda et al. 2009; Ikeda et al. 2011b). In a model experiment with simultaneous live-cell miRNA imaging using the mixture of ECHO probes with different fluorescence colors, each ECHO probe recognized the corresponding target miRNA in the cell and emitted the corresponding fluorescence color with a miRNA-specific emission wavelength.

Desmethyl TO dye unit has been developed for a pH-sensitive ECHO probe (Okamoto et al. 2013). The DNA probe doubly modified with desmethyl TO dye units showed effective on/off switching of fluorescence at pH 5–7, which is similar to the behavior of conventional ECHO probes. On the other hand, the fluorescence from the probe at higher pH (pH 8–10) was weak even in the presence of the complementary RNA strand, because acidic pH is important for the protonation and emission of desmethyl TO dye units.

**Fig. 3:**
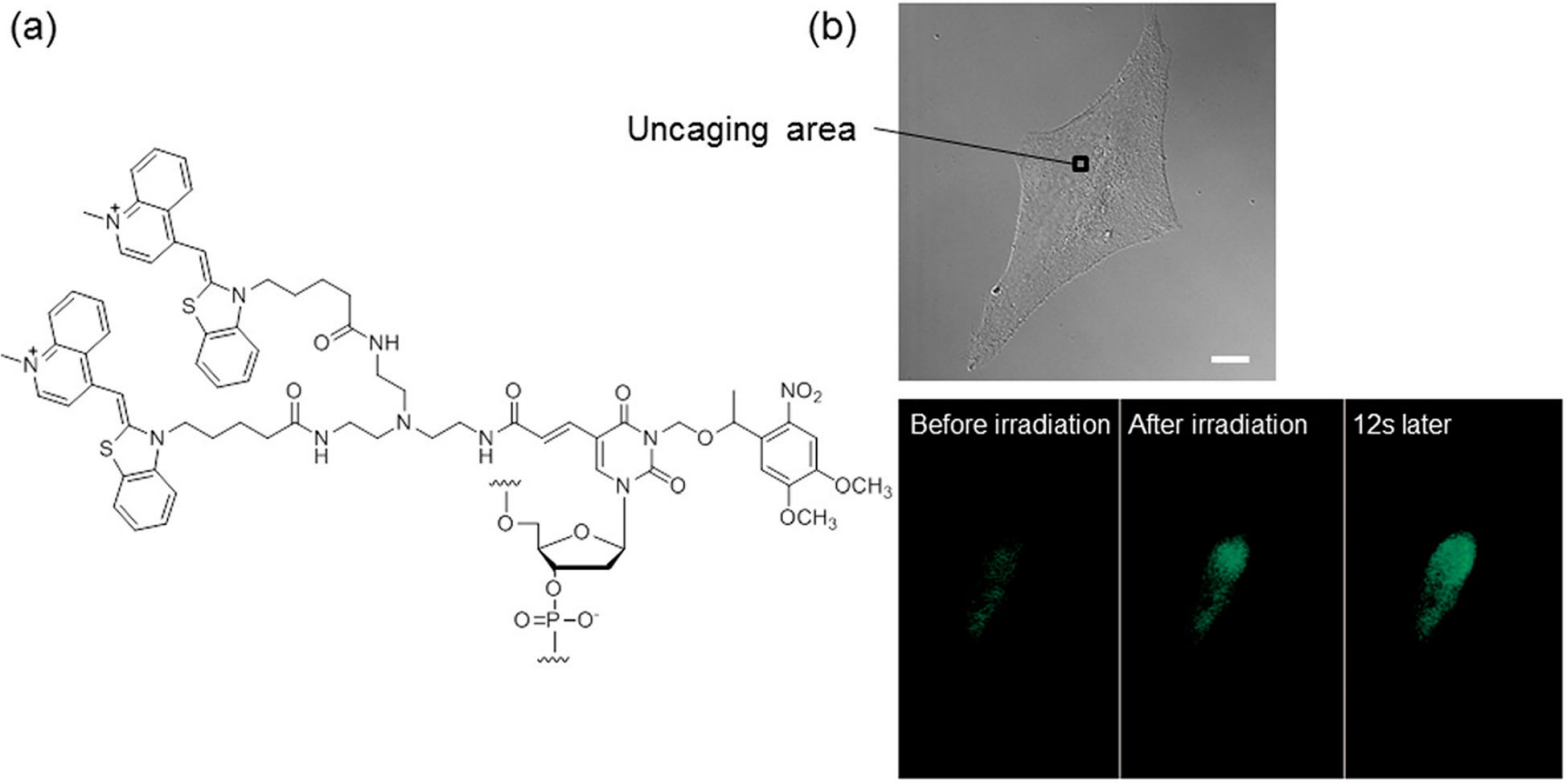
Pinpoint activation of caged ECHO probe. **a** Chemical structure; **b** Diffusion of the fluorescence after uncaging at an irradiation area (square) in the nucleus of the cell into which a caged ECHO probe was injected. Bar: 10 μm

Caged ECHO probes were also developed for pinpoint activation in a cell (Ikeda et al. 2011a). Caging technology is sometimes used for activation of substances in a spatiotemporal manner. The ECHO probe, protected by a photolabile nitrobenzyl unit, suppressed the function of the probes by decreasing the hybridization ability of probes (Fig. 3). After photolysis of the nitrobenzyl unit, the probes recovered the property of hybridization-sensitive fluorescence emission. Pinpoint uncaging of the caged ECHO probe in a living cell using a blue laser was applicable to visualization of subnuclear mRNA diffusion. The caged ECHO probe offers an alternative to the most popular area-specific photocontrolled fluorescence recovery after photobleaching assay or fluorescence loss in photobleaching assay.

Finally, we developed a high-resolution fluorescence RNA imaging method, ECHO-liveFISH, to visualize unmodified, native RNA at intranuclear foci in living mice and chicks (Oomoto et al. 2015). We combined ECHO-FISH probe technology with in vivo electroporation to achieve microscopic imaging in native tissues. This method enabled us to observe the spatial dynamics of individual ribonuclear foci, which are regulated by cellular transcriptional activity, at the single-cell level. Confirming the physiological relevance of this technique, the in vivo RNA labeling did not interfere with the function of the target RNA or cause noticeable cytotoxicity or perturbation of cellular behavior. With ECHO-liveFISH, we also detected the spatial dynamics demonstrated by the disintegration of 28S rRNA concentration upon actinomycin D treatment. The imaging result is consistent with the previous data in other species showing that the assembly of nucleoli is triggered by activation of rDNA transcription. Many advantages are offered by ECHO-liveFISH for in vivo RNA imaging, such as easy and effective delivery, negligible toxicity, lack of perturbation of cellular functions, stability in a living cellular environment, brightness, high signal-to-noise ratio, fast hybridization and photoactivation kinetics, reversible fluorescence emission, and data acquisition with standard confocal imaging systems.

## Methyl-specific fluorescent in situ hybridization

Transcription of DNA to RNA is well controlled by the epigenetic modification of DNA and histone proteins, independent of their primary sequences. One of epigenetic DNA modifications, methylation of cytosine (C), plays a crucial role in the regulation of gene expression (Jones and Wolffe 1999; Li 2002), and erroneous DNA methylation contributes to the etiopathogenesis of tumorigenesis and aging (Jones and Laird 1999; Robertson 2005). Much effort has gone into developing a simple reaction that can be used for the detection of intracellular 5-methylcytosine (5mC).

**Fig. 4:**
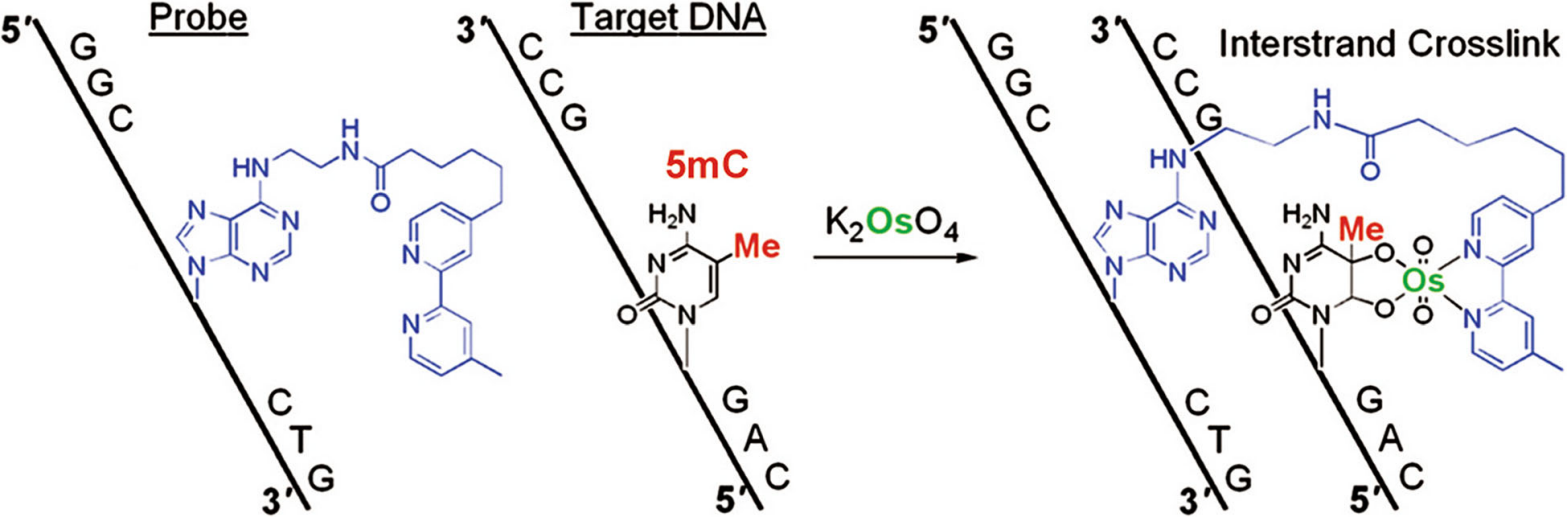
Crosslinking of ICON probe with the target 5mC in DNA by osmium complex formation

**Fig. 5:**
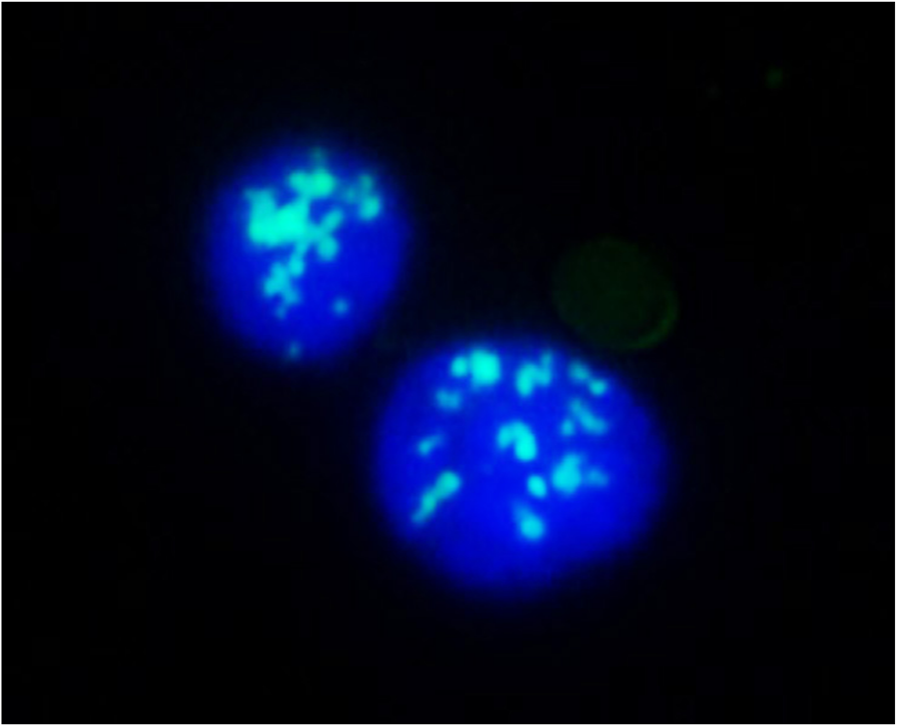
MeFISH image for major satellite region in mouse embryonic stem cells after osmium treatment and denaturation (green, ICON probe; blue, DAPI)

We developed an artificial DNA “ICON probe” for sequence-specific 5mC detection (Tanaka et al. 2007; Okamoto 2014). The chemistry of ICON probe is based on an interstrand complexation formed by osmium and nucleic acid (ICON). We tethered an adenine base forming a mismatched pair with 5mC to the bipyridine ligand required for osmium-centered complex formation. The formation of a 5mC/A mismatched pair causes partial disruption of π-stacking of the DNA duplex, and facilitates the osmium oxidation mediated by a bipyridine ligand at only the target 5mC C5–C6 double bond (Fig. 4). ICON technology is useful for identifying 5mC residues in target sequences at both the cellular and chromosomal levels. ICON probes modified with a fluorescent dye at the strand end can label only the target 5mC, without causing serious DNA damage as observed with bisulfite assays or without the problems associated with nonsequence-specific 5mC binding as observed with anti-5mC antibodies. We applied ICON technology to develop a novel method, named methylation-specific fluorescence in situ hybridization (MeFISH) by the collaboration with Sasaki group in Kyushu University. MeFISH assay is very effective for visualizing the DNA methylation status at specific sequences in individual nuclei or chromosomes (Li et al. 2013). For example, MeFISH was used to detect DNA methylation at centromeric and pericentromeric repeat sequences in both mouse and human cells. ICON probes were designed against major and minor satellite repeats of wild-type and *Dnmt*-TKO (*Dnmt*1, *Dnmt*3a, and *Dnmt*3b triple knock out) mouse embryonic stem cells, and two-color MeFISH was performed by using these probes (Fig. 5). On metaphase chromosomes, the major satellite probe displayed a strong signal near the centromeric end, and the minor satellite probe gave a doublet on sister chromatids at the centromeric end. In interphase nuclei, whereas the major satellite probe gave strong signals that were colocalized with the 4′,6-diamidino-2-phenylindole (DAPI)-dense regions, the minor satellite probe gave smaller spots at the periphery of the major satellite signals. The fluorescence signals from satellites were observed from both wild-type and *Dnmt*-TKO cells after the first FISH step, whereas the specific retention of ICON signals in wild-type, but not in *Dnmt*-TKO cells was clear after treatment with osmium and subsequent removal of noncrosslinked probes.

Furthermore, MeFISH was applied to detect methylation at human satellite repeat sequences. Human classical satellites 2 and 3 and alpha satellite are generally highly methylated in somatic cells from healthy individuals, but they are hypomethylated in those from patients with immunodeficiency, centromeric instability, and facial anomalies (ICF) syndrome. The syndrome is classified into two types with different hypomethylation levels at satellites. When we examined MeFISH, the control cells showed fluorescent signals at both classical satellite and alpha satellite. In type 1 ICF cells, the classical satellite signals were very low although the alpha satellite MeFISH signals were detectable. On the other hand, signals were undetectable at both satellites in type 2 ICF cells under the same photographic exposure conditions. The faint MeFISH signals were detected even in the ICF specimens, which was consistent with the facts that these sequences are hypomethylated, but not completely unmethylated, in ICF cells (Jiang et al. 2005; Jeanpierre et al. 1993; Brun et al. 2011).

Furthermore, the collaboration with Abe group in RIKEN adapted MeFISH to the study of intact embryos, and established a method called “whole-mount MeFISH” (Shiura et al. 2014). The DNA methylation status of satellite repeats in developing mouse primordial germ cells, in which global DNA demethylation is known to take place, was examined and a result that was consistent with previous findings was obtained. We also combined whole-mount MeFISH with immunostaining or FISH techniques. These combined whole-mount MeFISH methods enabled the simultaneous visualization of DNA methylation and protein or RNA expression at single-cell resolution without destroying embryonic and nuclear structures. This whole-mount MeFISH technique should facilitate the study of the dynamics of DNA methylation status during embryonic development with unprecedented resolution.

## Conclusions

Fluorescent imaging of intracellular nucleic acids has made great progress over the last few decades. In this mini-review, we introduced a couple of unique fluorescent nucleic acid probes for effective microscopic imaging of intracellular RNA and methylated DNA. The ECHO probe technology, one of the TO probes, made it possible to detect the target nucleic acid and to analyze both the spatiotemporal dimensions of the diverse RNA dynamics in a living cell. The hybridization sensitivity of fluorescence emission from ECHO probes is a great advantage in live cell imaging. Further expansion of the diversity of ECHO probes should lead to further improvements in the methodology of nucleic acid imaging. The ICON probe technology and its application to MeFISH were also described in this mini-review. The sequence-specific formation of a stable osmium complex by ICON probes made the fluorescence imaging of DNA methylation at the target cytosine in a cell possible. This new concept of DNA methylation imaging technology at a single cell level will be the starting point for an epoch-making methylation-imaging assay, which will supersede conventional methods.

## Data Availability

The datasets used and/or analyzed during the current study are available from the corresponding author on reasonable request.
